# H3K27ac acts as a molecular switch for doxorubicin-induced activation of cardiotoxic genes

**DOI:** 10.1186/s13148-024-01709-8

**Published:** 2024-07-16

**Authors:** Yu Hong, Xinlan Li, Jia Li, Qiuyi He, Manbing Huang, Yubo Tang, Xiao Chen, Jie Chen, Ke-Jing Tang, Chao Wei

**Affiliations:** 1 Department of Pharmacy, The First Affiliated Hospital, Sun Yat-Sen University, Guangzhou, China; 2 Guangdong Provincial Key Laboratory of New Drug Design and Evaluation, School of Pharmaceutical Sciences, Sun Yat-Sen University, Guangzhou, China; 3 Department of Critical Care Medicine, Guangdong Provincial People’s Hospital, Guangdong Academy of Medical Sciences, Guangzhou, China; 4 Zhongshan School of Medicine, Sun Yat-Sen University, No.74 Zhongshan Rd.2, Guangzhou, 510080 China

**Keywords:** Histone modification, Broad H3K27ac, Dox-induced cardiotoxicity, DNA damage

## Abstract

**Background:**

Doxorubicin (Dox) is an effective chemotherapeutic drug for various cancers, but its clinical application is limited by severe cardiotoxicity. Dox treatment can transcriptionally activate multiple cardiotoxicity-associated genes in cardiomyocytes, the mechanisms underlying this global gene activation remain poorly understood.

**Methods and results:**

Herein, we integrated data from animal models, CUT&Tag and RNA-seq after Dox treatment, and discovered that the level of H3K27ac (a histone modification associated with gene activation) significantly increased in cardiomyocytes following Dox treatment. C646, an inhibitor of histone acetyltransferase, reversed Dox-induced H3K27ac accumulation in cardiomyocytes, which subsequently prevented the increase of Dox-induced DNA damage and apoptosis. Furthermore, C646 alleviated cardiac dysfunction in Dox-treated mice by restoring ejection fraction and reversing fractional shortening percentages. Additionally, Dox treatment increased H3K27ac deposition at the promoters of multiple cardiotoxic genes including *Bax*, *Fas* and *Bnip3*, resulting in their up-regulation. Moreover, the deposition of H3K27ac at cardiotoxicity-related genes exhibited a broad feature across the genome. Based on the deposition of H3K27ac and mRNA expression levels, several potential genes that might contribute to Dox-induced cardiotoxicity were predicted. Finally, the up-regulation of H3K27ac-regulated cardiotoxic genes upon Dox treatment is conservative across species.

**Conclusions:**

Taken together, Dox-induced epigenetic modification, specifically H3K27ac, acts as a molecular switch for the activation of robust cardiotoxicity-related genes, leading to cardiomyocyte death and cardiac dysfunction. These findings provide new insights into the relationship between Dox-induced cardiotoxicity and epigenetic regulation, and identify H3K27ac as a potential target for the prevention and treatment of Dox-induced cardiotoxicity.

## Background

Chemotherapy has been a standard therapeutic approach in clinics for various cancers, which utilizes cytotoxic chemical drugs to induce the necrosis of tumor cells [1, 2]. However, many chemotherapy drugs have serious side effects, which limit their widespread usage [1, 3, 4]. Doxorubicin (Dox), an anthracycline drug, is widely used for treating various tumors, including leukemia, lymphomas, breast cancer, lung cancer, and other solid tumors [5, 6]. However, the clinical application of Dox is limited due to its dose-dependent cardiotoxicity, which can lead to ventricular wall dysfunction and progressive heart failure [5, 7, 8].

Several mechanisms have been proposed for Dox-induced cardiotoxicity, including apoptosis and oxidative stress, which are triggered by the upregulation of *Bax* and *Fas* [9–13]. Histone modifications, such as methylation, acetylation, and ubiquitination, epigenetically regulate gene expression by remodeling chromatin and activating or repressing transcription [14, 15]. Studies show that Dox treatment decreases the level of histone deacetylases (HDACs) [16], this raises the possibility that Dox may regulate the expression of numerous genes through global histone acetylation, ultimately leading to adverse effects on the myocardium.

H3K27 acetylation (H3K27ac) is an epigenetic marker of active gene promoters and enhancers [17–19]. In this study, we observed that Dox treatment increased the deposition of H3K27ac across genome in cardiomyocytes, resulting in the upregulation of genes associated with cardiotoxicity. C646, a small molecule inhibitor of histone acetyltransferase [20], reduced H3K27ac enrichment in the nuclei of cardiomyocytes and alleviated Dox-induced cardiotoxicity. Thus, H3K27ac acts as a molecular switch for the activation of cardiotoxicity genes induced by Dox and represents a promising target for improving the therapeutic efficacy of Dox while minimizing cardiotoxicity.

## Results

### Dox treatment led to H3K27ac accumulation in cardiomyocytes

**Fig. 1:**
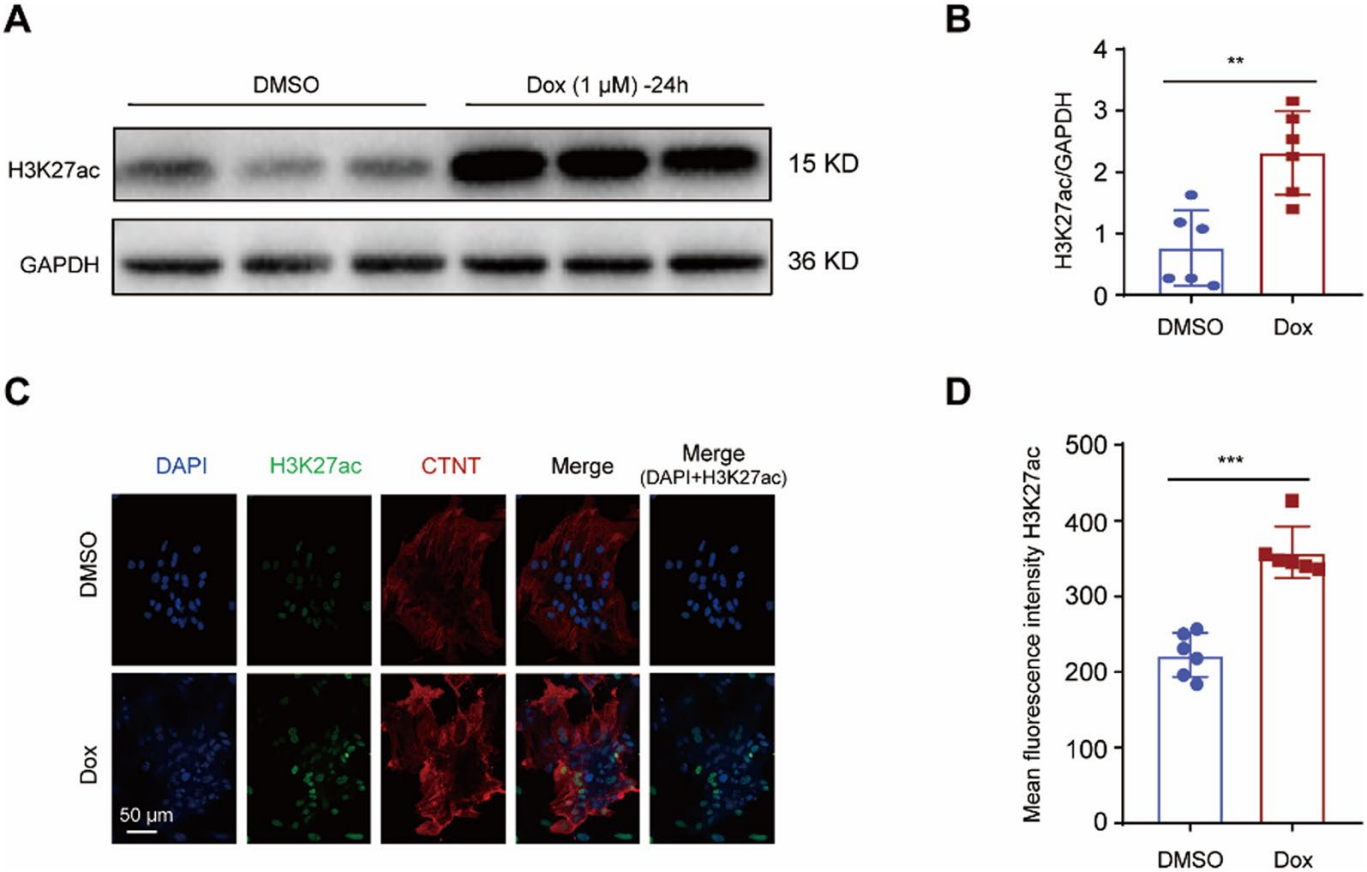
Dox treatment led to H3K27ac accumulation in cardiomyocytes. **A**, **B** Representative western blot (**A**) and statistical data (**B**) showing the levels of H3K27ac from the cardiomyocytes treated with Dox for 24 h, n = 6 per group. **C**, **D** Representative immunostaining image (**C**) and mean fluorescence intensity (**D**) for H3K27ac (green) in the cardiomyocytes treated with Dox for 24 h; CTNT denotes cardiac troponin T (red); Nuclei were stained with DAPI (blue)

H3K27ac promotes gene transcription by regulating promoter and enhancer activity [17–19]. To investigate whether Dox affects H3K27ac accumulation in cardiomyocytes, we treated neonatal rat cardiomyocytes (NRCMs) with 1 µM Dox for 24 h and analyzed the levels of H3K27ac using a specific antibody. The total levels of H3K27ac were significantly higher in the Dox-treated cells compared to the control cells treated with DMSO (Fig. 1A, B). Additionally, immunostaining of the cardiomyocytes (CTNT-positive) revealed significantly higher intensity of H3K27ac in the Dox-treated cells compared to the DMSO-treated group (Fig. 1C, D). Overall, Dox leads to increased accumulation of H3K27ac in cardiomyocytes, suggesting a potential role for H3K27ac in Dox-induced cardiotoxicity.

### Reducing H3K27ac accumulation via C646 ameliorated Dox-induced DNA damage in cardiomyocytes

**Fig. 2:**
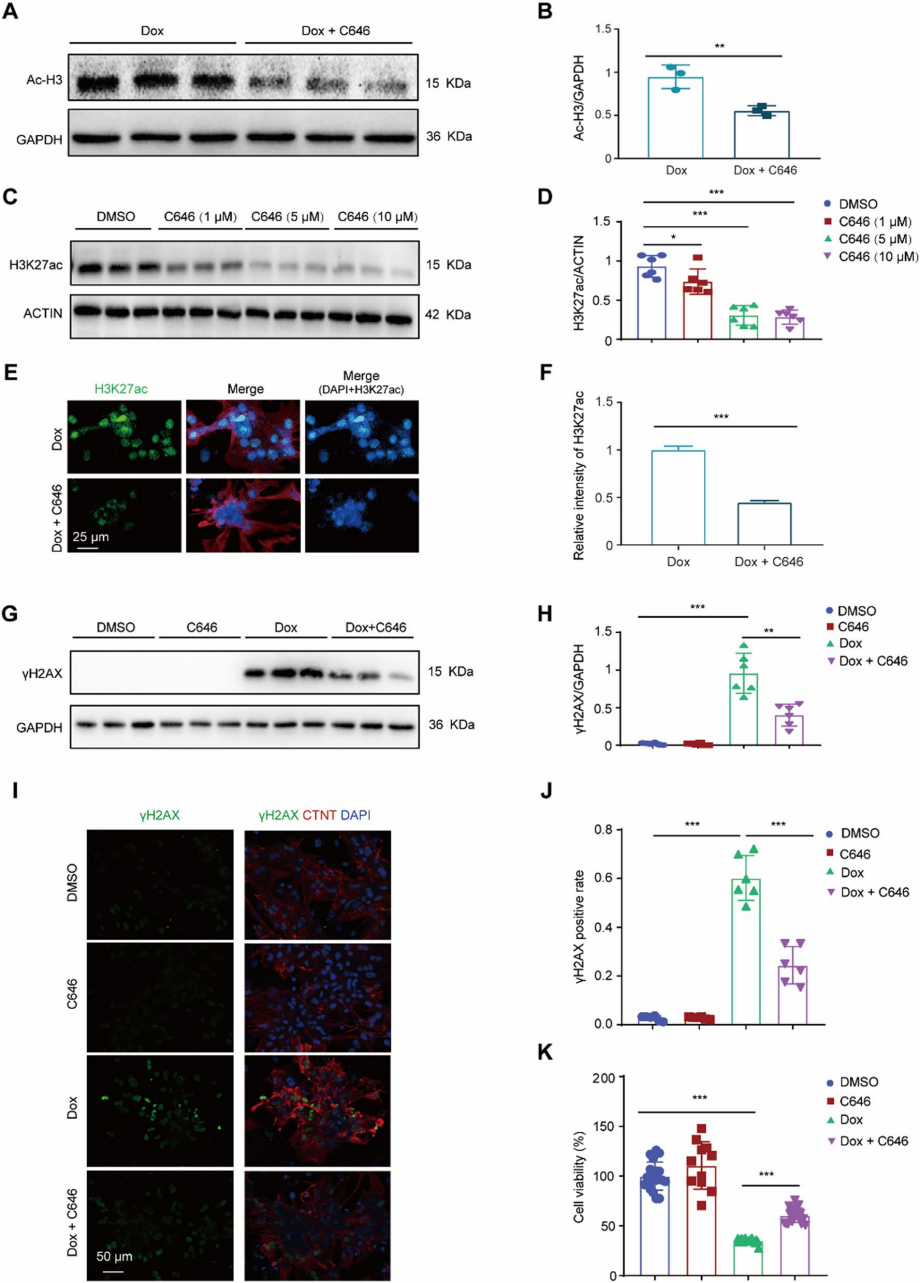
Reducing H3K27ac accumulation via C646 ameliorated Dox-induced DNA damage in cardiomyocytes. **A**, **B** Representative western blot (**A**) and statistical data (**B**) showing the levels of Ac-H3 from the cardiomyocytes treated with Dox or Dox with C646 for 24 h. n = 3 per group. **C**, **D** Representative western blot (**C**) and statistical data (**D**) showing the levels of H3K27ac from the cardiomyocytes treated with increasing concentrations of C646 ranging from 0 to 10 μM for 24 h. n = 6 per group. **E**, **F** Representative immunostaining image (**E**) and relative fluorescence intensity (**F**) for H3K27ac (green) in the cardiomyocytes treated with Dox or Dox with C646 for 24 h; red field denotes cardiac troponin T; Nuclei were stained with DAPI (blue). **G**, **H** Representative western blot (**G**) and statistical data (**H**) showing the levels of γH2AX from Dox-treated cardiomyocytes with or without C646 for 24 h. n = 6 per group. **I**, **J** Representative immunostaining image and analysis for γH2AX (green) in Dox-treated cardiomyocytes with or without C646 for 24 h. **K** CCK-8 assay revealed cell viability after Dox treatment with or without C646, respectively

We next investigated whether reducing H3K27ac accumulation can alleviate Dox-induced cardiotoxicity. P300 is a histone acetyltransferase involved in the deposition of H3K27ac [21]. C646 is an inhibitor of P300, and has been shown to effectively reduce the acetylation of histone H3 (Ac-H3) [22, 23], which conclusion was validated in Dox-induced cardiomyocytes (Fig. 2A, B). In addition, C646 is also reported to reduce H3K27ac accumulation [24]. The dosage of C646 which is able to effectively reduce H3K27ac level was firstly investigated. NRCMs were isolated and treated with various concentrations (0 to 10 μM) of C646 for 24 h in vitro. At a dose of 10 μM, C646 significantly reduced H3K27ac levels in NRCMs (Fig. 2C, D). In addition, C646 is also able to reduce H3K27ac levels in the nucleus of Dox-treated NRCMs (Fig. 2E, F).

Next, we examined the impact of C646 on Dox-induced DNA damage by analyzing the levels of DNA damage marker γH2AX [25]. Cells treated with Dox alone exhibited increased γH2AX accumulation, which was significantly inhibited by C646 (Fig. 2G, H). Moreover, Dox treatment led to an increase in the proportion of γH2AX-positive cells, which was reduced by C646 (Fig. 2I, J). Additionally, C646 restored cell viability in cells treated with Dox, as determined by the CCK8 assay (Fig. 2K). Altogether, C646 reversed H3K27ac accumulation and protected cardiomyocytes from Dox-induced DNA damage.

### Decreased H3K27ac accumulation attenuated Dox-induced apoptosis in cardiomyocytes

**Fig. 3:**
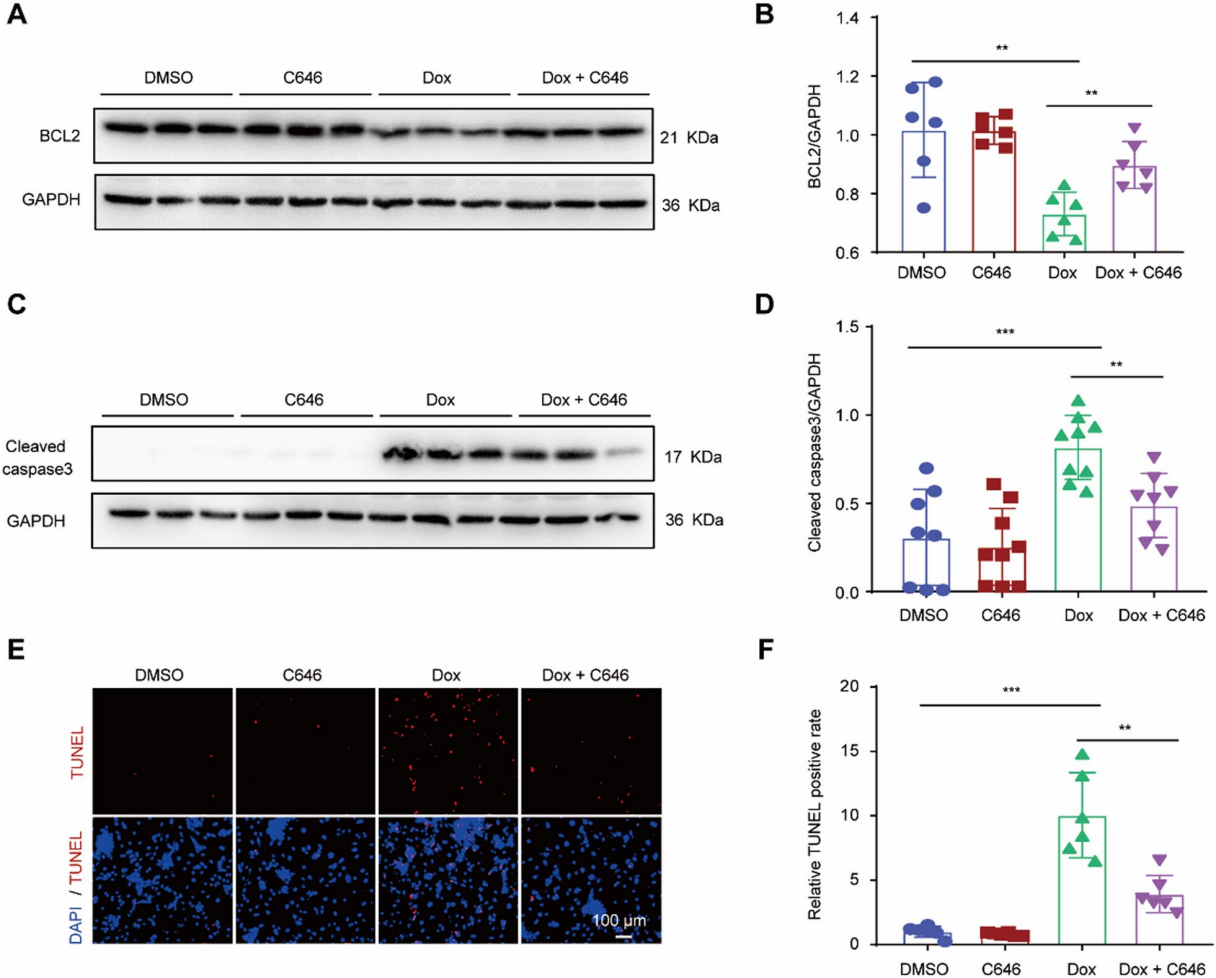
Decreased H3K27ac accumulation attenuated Dox-induced apoptosis in cardiomyocytes. **A**, **B** Representative western blot (**A**) and statistical data (**B**) showing the levels of BCL2 from Dox-treated cardiomyocytes with or without C646 for 24 h. n = 6 per group. **C**, **D** Representative western blot (**C**) and statistical data (**D**) showing the levels of cleaved caspase3 from Dox-treated cardiomyocytes with or without C646 for 24 h. n values are 8, 9, 9, 8, respectively. **E**, **F** Representative images (**E**) and statistical data (**F**) of TUNEL-positive cells (red) from Dox-treated cardiomyocytes with or without C646 for 24 h. n = 6 per group

To investigate whether H3K27ac accumulation mediates Dox-induced apoptosis in NRCMs, we analyzed the impact of H3K27ac inhibition on the expression levels of apoptosis-related proteins. NRCMs were treated with DMSO, C646, Dox and Dox + C646, then the expression of both anti-apoptotic protein BCL2 and pro-apoptotic protein cleaved caspase3 were detected [26, 27]. Dox treatment resulted in a significant decrease in BCL2 levels and a significant increase in cleaved caspase3 protein levels in cultured cardiomyocytes, both of which were rescued by C646 (Fig. 3A–D). Furthermore, Dox increased the number of TUNEL-positive cardiomyocytes, which was reduced by C646 (Fig. 3E, F). Consequently, decreased H3K27ac accumulation attenuated Dox-induced apoptosis in cardiomyocytes.

### H3K27ac suppression protected against Dox-induced dysfunction of heart

**Fig. 4:**
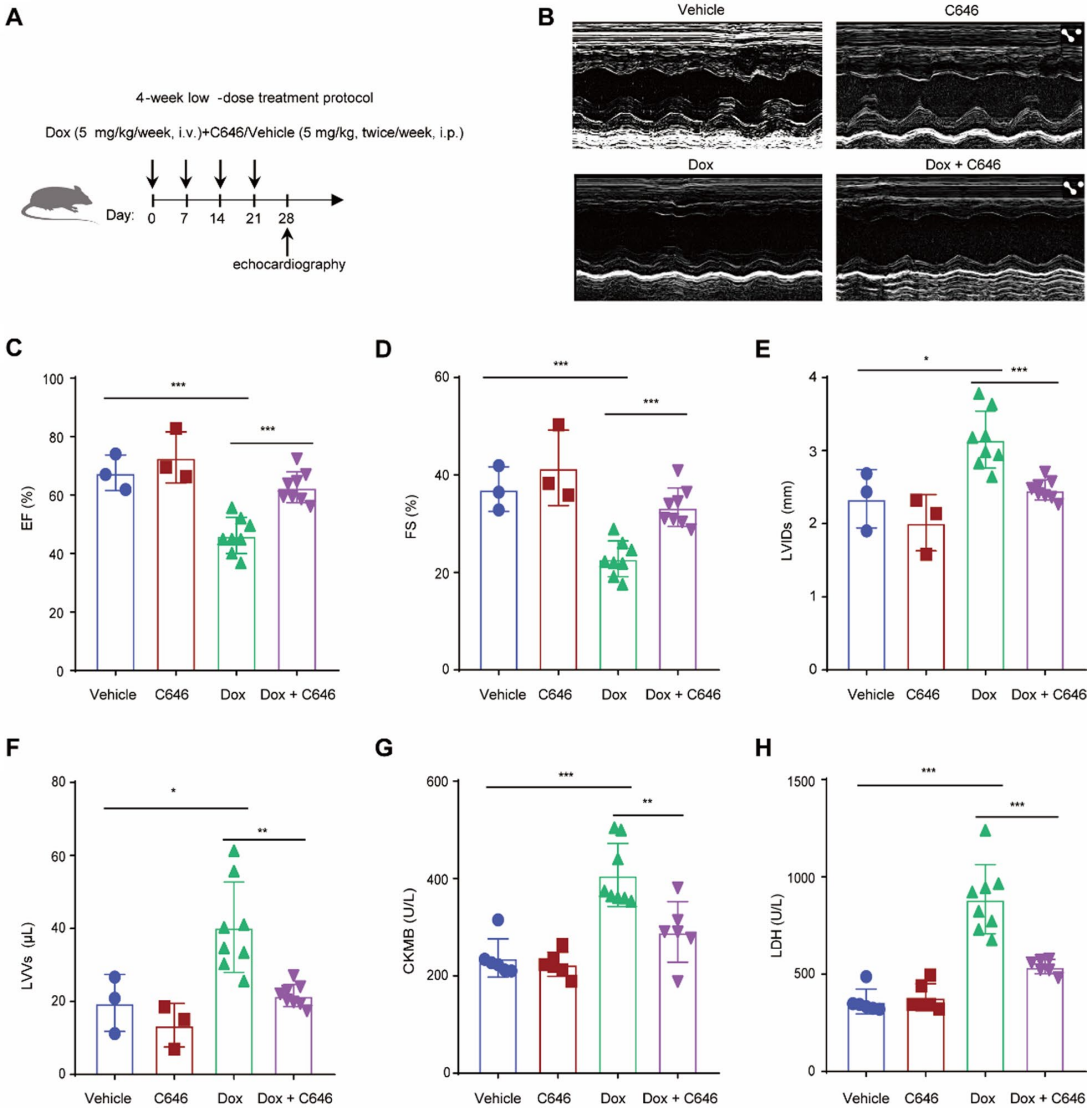
H3K27ac suppression protected the hearts from the functional impairment caused by Dox in mice. **A** Experimental overview of present animal experiment, “i.v.” denotes Intravenous injection, “i.p.” denotes Intraperitoneal injection. **B** Representative echocardiographic images of Dox-treated mice heart with or without C646 for 4 weeks. **C**, **D** Ejection fraction (**C**) and fractional shortening (**D**) data of Dox-treated mice heart with or without C646 for 4 weeks. **E**, **F** Left ventricular internal diameter at end systole (**E**) and left ventricular volume at end systole of Dox-treated mice heart with or without C646 for 4 weeks. **G**, **H** CKMB (creatine kinase MB) (**G**) and LDH (lactic dehydrogenase) (**H**) activity from serum of mice, which were treated with Dox combined with or without C646 for 4 weeks

To investigate the role of H3K27ac in heart damage in vivo, we established a mouse model of Dox-induced cardiac injury via administering Dox to wild-type mice via intravenous injection, with the control group receiving saline. Following this, we gave the mice intraperitoneal injections of either C646 or the drug solvent (Fig. 4A). The cardiac function in different groups was evaluated by ejection fraction (EF), fractional shortening (FS), left ventricular internal diameter at end systole (LVIDs) and left ventricular volume at end systole (LVVs). The results showed that after 4 weeks of treatment, the Dox-treated mice exhibited a significant decrease in EF and FS, along with increased LVIDs and LVVs compared to the vehicle control mice (Fig. 4B–F). C646 treatment prevented Dox-induced decrease of EF and FS, and also prevented Dox-induced increase of LVIDs and LVVs (Fig. 4B–F). This indicated that Dox caused cardiac dysfunction, and C646 restored the cardiac function in the Dox-treated mice. Next, the biochemical indices of heart failure including LDH and CKMB were measured, to assess the cardiotoxic effects of Dox. Consistent with the echocardiography results, Dox treatment significantly increased the levels of CKMB and LDH (Fig. 4G, H). These levels were restored to near normal in the mice that received additional treatment with C646 (Fig. 4G, H), confirming the cardioprotective effect of C646. Altogether, our findings suggest that suppressing H3K27ac with C646 can protect against Dox-induced cardiotoxicity in vivo.

### Dox increased broad H3K27ac deposition at the promoters of cardiotoxic genes

**Fig. 5:**
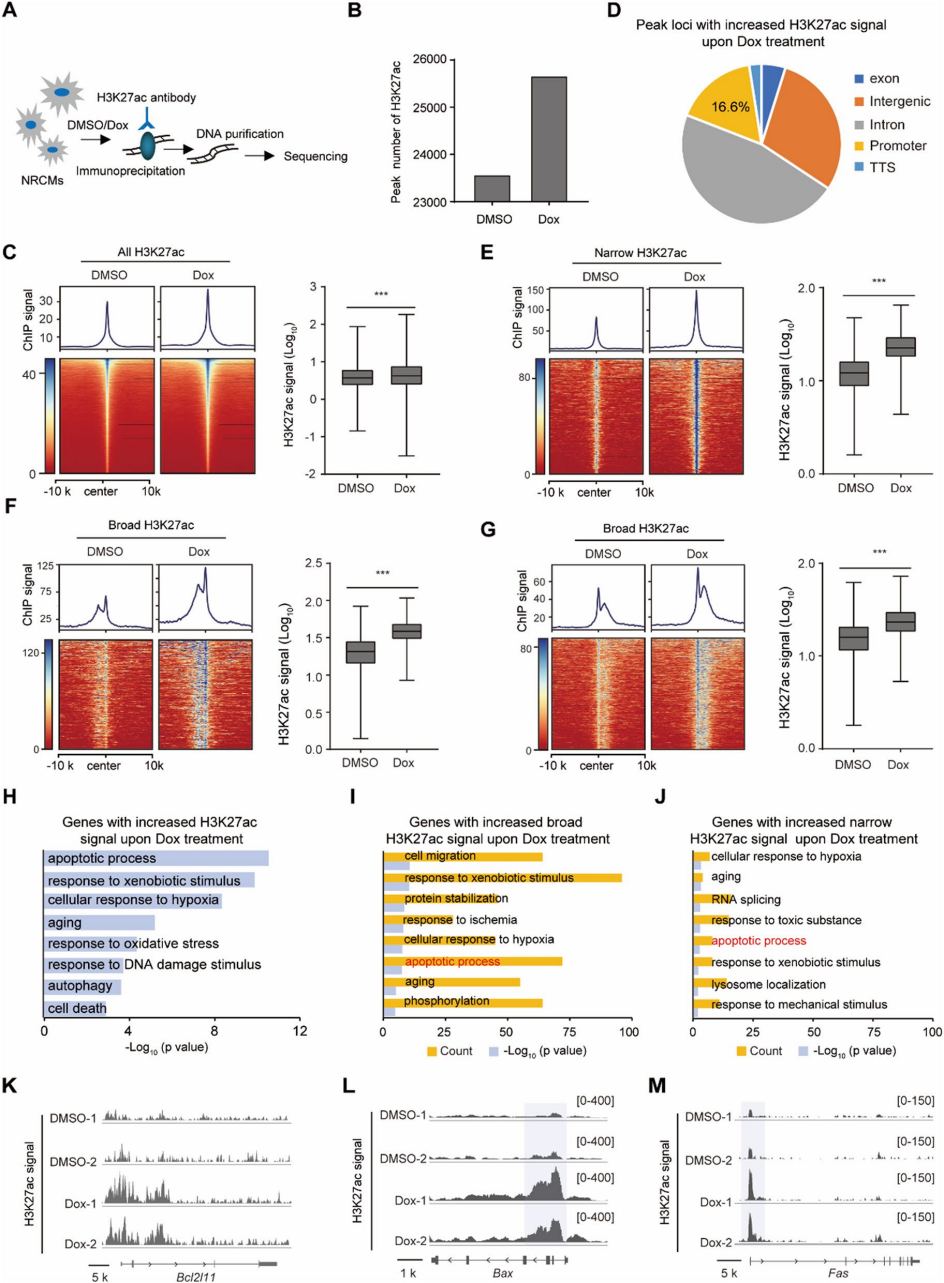
Dox increased broad H3K27ac deposition at the promoters of cardiotoxicity-related genes. **A** Overview of H3K27ac CUT&Tag experiment. **B** Peak number of H3K27ac in DMSO and Dox group. **C** Heatmap and quantification showing the global H3K27ac signal in DMSO and Dox group, two-tailed Wilcoxon test. **D** The distribution of signal-increased H3K27ac peak upon Dox treatment. **E** Heatmap and quantification showing the increased ChIP signal at narrow H3K27ac peak loci in DMSO and Dox group, two-tailed Wilcoxon test. **F**, **G** Heatmap and quantification showing the increased ChIP signal at broad H3K27ac peak loci in DMSO and Dox group, two-tailed Wilcoxon test. **H** Gene ontology analysis showing the biological processes for all the genes with increased H3K27ac signal upon Dox treatment. **I**, **J** Gene ontology analysis showing the biological processes for the genes with increased broad (**I**) and narrow H3K27ac (**J**) signal upon Dox treatment, and the apoptotic process was highlighted. **K**–**M** The representative regions showing the H3K27ac signal around *Bcl2l11*, *Bax* and *Fas* genes between the DMSO and Dox group with two replicates

To further investigate the relationship between genome-wide changes in H3K27ac upon Dox treatment and cardiotoxicity-related genes, we performed H3K27ac CUT&Tag experiments in NRCMs treated with either Dox or DMSO (Fig. 5A). Dox treatment increased the total number of H3K27ac peaks (Fig. 5B), as well as the global H3K27ac signal (Fig. 5C). Specifically, 9922 loci showed increased H3K27ac signal upon Dox treatment, with 46.6% and 16.6% of them localized at intron and promoter regions, respectively. 29.5% and 4.8% were found in intergenic and exon regions, respectively (Fig. 5D).

Among the increased H3K27ac peaks, a subset of them displayed the typical narrow peaks (Fig. 5E). Genes marked with the broad epigenetic domain have clinical potential as biomarkers for patient stratification [28], and we observed majority of loci with increased broad H3K27ac signal upon Dox treatment (Fig. 5F, G). Then the biological processes of genes that had increased narrow or broad H3K27ac peaks at their promoters were analyzed. Overall, genes with increased H3K27ac signal after Dox treatment were enriched in multiple terms associated with cardiotoxicity, including apoptotic process and cell death (Fig. 5H). However, these genes were more likely to be occupied by increased broad H3K27ac peaks rather than narrow H3K27ac peaks (Fig. 5I, J). For example, after Dox treatment, 72 genes associated with apoptotic process were occupied by increased broad H3K27ac peaks, while only 8 apoptotic genes were occupied by increased narrow H3K27ac peaks (Fig. 5I, J). As three examples, BCL2L11 is a pro-apoptosis protein [29]*,* Dox increased the deposition of H3K27ac around the transcription start site (TSS) of *Bcl2ll1* for nearly 15 kb (Fig. 5K). *Bax* and *Fas* showed a positive correlation with the severity of Dox-induced cardiotoxicity [30, 31]. DOX increased the deposition of H3K27ac around the TSS of *Bax* and *Fas* for nearly 6 kb and 5 kb, respectively (Fig. 5L, M). Taken together, Dox increased H3K27ac deposition at promoters of genes involved in cardiotoxicity-associated pathway.

### Dox up-regulated the expression of cardiotoxicity-related genes

**Fig. 6:**
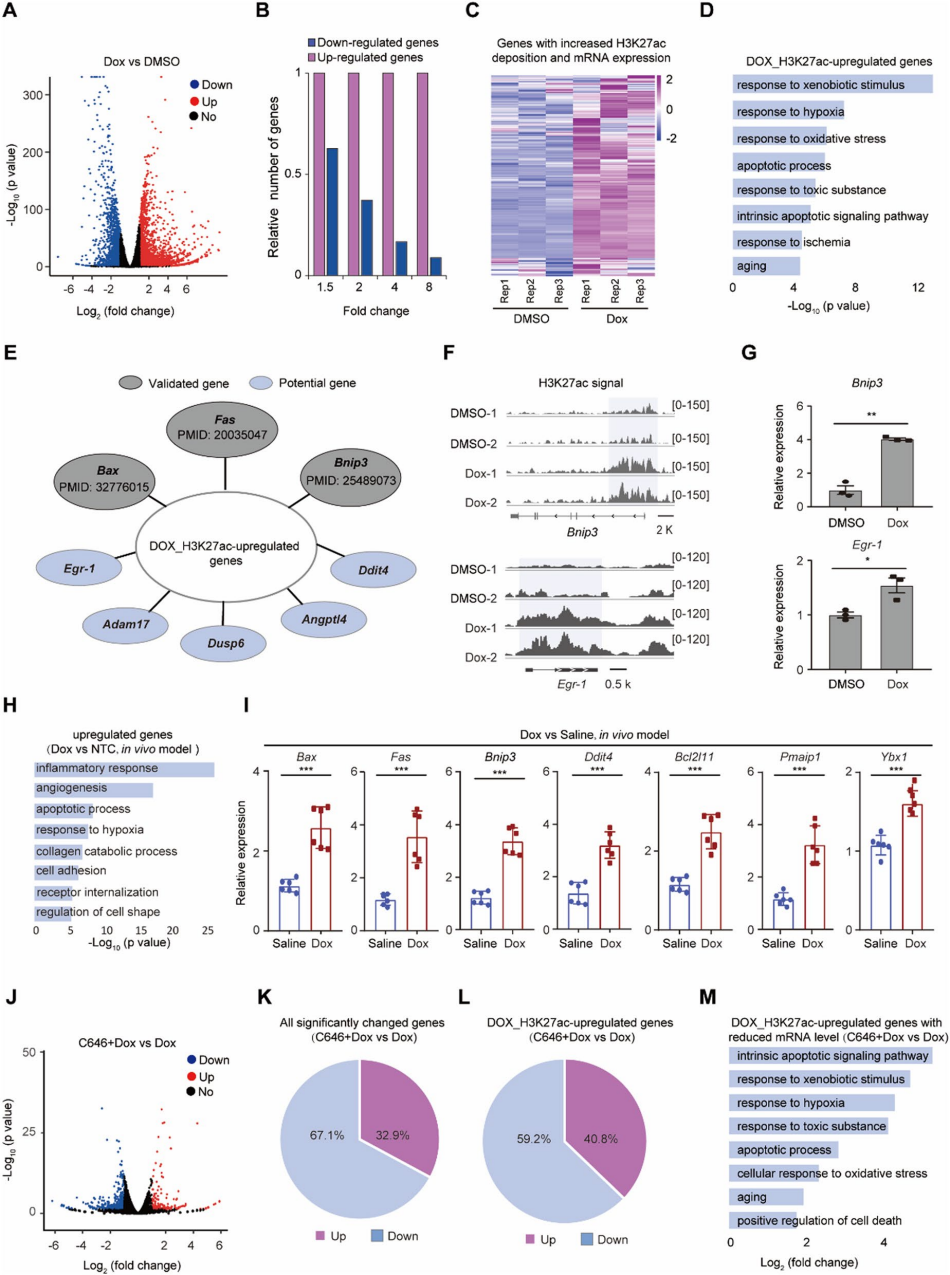
Dox treatment upregulated cardiotoxicity-related genes. **A** Volcano plot showing the expression alteration of genes in rat cardiomyocytes upon Dox treatment. **B** Histogram showing the number of down-regulated and up-regulated genes with different fold change in cardiomyocytes upon Dox treatment. **C** Heatmap showing the genes with both increased H3K27ac signal and increased mRNA expression in rat cardiomyocytes upon Dox treatment. **D** Gene ontology analysis showing the biological processes for the genes with both increased H3K27ac signal and increased mRNA expression in rat cardiomyocytes upon Dox treatment. **E** The representative genes with both increased H3K27ac signal and increased mRNA expression in cardiomyocytes upon Dox treatment, several genes are reported to be associated with Dox-induced cardiotoxicity including *Fas*, *Bax* and *Bnip3*, other genes are potentially associated with cardiotoxicity. **F**, **G** H3K27ac signal nearby *Bnip3 and Egr-1* (**F**) and their mRNA expression (**G**) in cardiomyocytes upon Dox treatment with two replicates. **H** Gene ontology analysis showing the biological processes for the upregulated genes in cardiomyocytes from an in vivo model upon Dox treatment (GSE223698). **I** Expression of apoptotic and cell death associated genes in cells from mouse heart upon Dox treatment for 24 h. **J** Compared to the Dox group, volcano plot showing the expression alteration of genes in cardiomyocytes upon Dox + C646 treatment. **K** Compared to the Dox group, the percentages of down-regulated and up-regulated genes after Dox + C646 treatment. **L** Compared to the Dox group, the percentage of down-regulated and up-regulated genes among Dox_H3K27ac-upregulated genes after Dox + C646 treatment. **M** Gene ontology analysis showing the biological processes for the Dox_H3K27ac-upregulated genes with decreased mRNA expression in cardiomyocytes upon Dox + C646 treatment

To verify whether Dox-induced H3K27ac deposition contributes to transcription alterations, we analyzed the transcriptomes of Dox-treated cardiomyocytes by RNA-seq. Both up-regulated and down-regulated genes were observed (Fig. 6A). When using a fold change cutoff ranging from 1.5 to 8, the total number of upregulated genes was higher than that of downregulated genes, particularly for genes with a fold change of 8 (Fig. 6B). Integrating the H3K27ac CUT&Tag data with the RNA-seq data from NRCMs after Dox treatment, we found that the majority of genes with increased H3K27ac signal were upregulated (referred to as Dox_H3K27ac-upregulated genes, Fig. 6C). Gene ontology (GO) analysis revealed that these genes were enriched in terms associated with cardiotoxicity, including the apoptotic process (Fig. 6D), further supporting the role of Dox-induced H3K27ac accumulation in cardiotoxicity. Notably, a subset of these genes, such as *Bax*, *Fas* and *Bnip3*, have been previously implicated in Dox-induced cardiotoxicity [9, 31–33] (Fig. 6E). Additionally, several Dox_H3K27ac-upregulated genes including *Egr-1, Adam17*, *Dusp6*, *Ddit4* and *Angptl4* might potentially contribute to Dox-induced cardiotoxicity (Fig. 6E), since they have been proved to be associated with the progression of cardiovascular disease [34–42]. Two examples are BNIP3, which mediates Dox-induced cardiomyocyte pyroptosis [33], and EGR-1, which promotes cardiomyocyte apoptosis [34]. DOX significantly increased H3K27ac signal around the TSS of *Bnip3* and *Egr-1*, with a broad distribution spanning approximately 10 kb and 4.5 kb, respectively (Fig. 6F). Both *Bnip3* and *Egr-1* were significantly up-regulated upon Dox treatment (Fig. 6G). In vivo, compared to the control group, the expression of 64 apoptotic genes was significantly upregulated in cardiomyocytes from Dox-treated hearts (Fig. 6H). In addition, expression of apoptotic and cell death associated genes, including Dox_H3K27ac-upregulated genes such as *Bax*, *Fas*, *Bnip3* and *Ddit4*, also significantly up-regulated in Dox-treated hearts (Fig. 6I). Thus, Dox increased the deposition of H3K27ac and the mRNA expression of genes associated with cardiotoxicity in cardiomyocytes.

Next, we investigated whether reducing H3K27ac accumulation by C646 would decrease the expression of Dox-induced cardiotoxicity-related genes, and performed RNA-seq in Dox-treated NRCMs with or without C646 treatment. Compared to Dox alone group (Dox group), the combined treatment of C646 with Dox (C646 + Dox group) could both up-regulated and down-regulated numerous genes (Fig. 6J). However, the number of down-regulated genes is obviously higher than that of up-regulated genes (67.1% vs 32.9%) (Fig. 6K), as well as the Dox_H3K27ac-upregulated genes (59.2% down-regulated genes vs 40.8% up-regulated genes) (Fig. 6L). In addition, the Dox_H3K27ac-upregulated genes with reduced mRNA expression were also enriched at apoptotic and cell death terms (Fig. 6M). Thus, C646 treatment reduced the expression of Dox_H3K27ac-upregulated genes involved in cardiotoxicity-related pathways.

In conclusion, Dox treatment increased the deposition of H3K27ac and mRNA expression of genes associated with cardiotoxicity, and their expression was restored upon reducing H3K27ac accumulation through C646 treatment.

### H3K27ac-regulated cardiotoxic genes were conservatively up-regulated upon Dox treatment across species

**Fig. 7:**
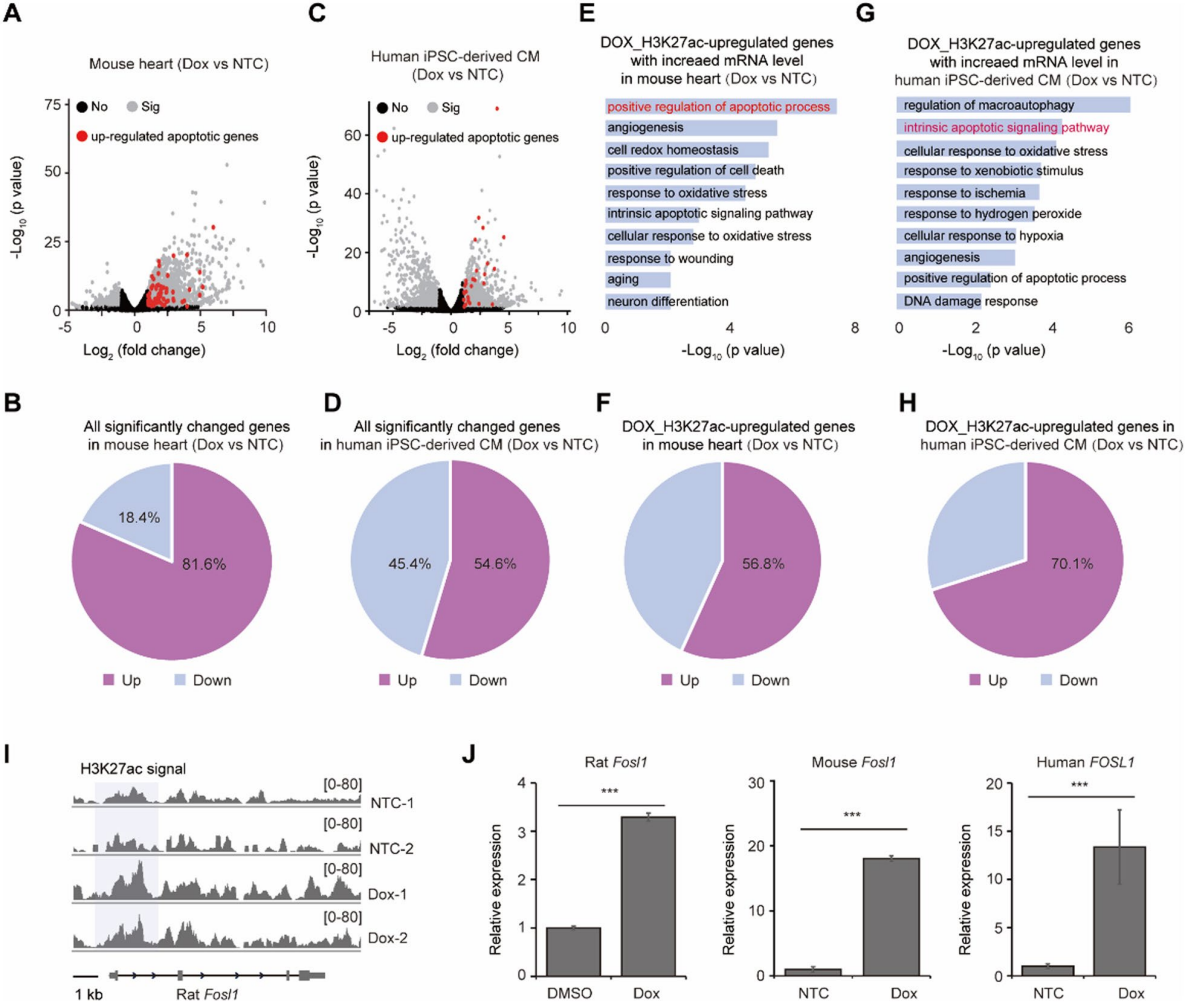
H3K27ac-regulated cardiotoxic genes were conservatively up-regulated upon Dox treatment across species. **A**, **B** Volcano plot showing the expression alteration of genes (**A**) and their percentages (**B**) in mouse heart (GSE226116) upon Dox treatment; black points denote the genes without significant change (No), gray points denote the genes with significant change (Sig), red points denote the apoptotic genes with significant up-regulation. **C**, **D** Volcano plot showing the expression alteration of genes (**C**) and their percentages (**D**) in human iPSC-derived cardiomyocytes (GSE230638) upon Dox treatment. CM denotes cardiomyocytes; black points denote the genes without significant change (No), gray points denote the genes with significant change (Sig), red points denote the apoptotic genes with significant up-regulation. **E** Gene ontology analysis showing the biological processes for the Dox_H3K27ac-upregulated genes with increased mRNA expression in mouse heart upon Dox treatment. **F** The percentage of down-regulated and up-regulated genes among Dox_H3K27ac-upregulated genes in mouse heart after Dox treatment. **G** Gene ontology analysis showing the biological processes for the Dox_H3K27ac-upregulated genes with increased mRNA expression in human iPSC-derived cardiomyocytes. **H** The percentage of down-regulated and up-regulated genes among Dox_H3K27ac-upregulated genes in human iPSC-derived cardiomyocytes after Dox treatment. **I** H3K27ac signal nearby *Fosl1* in rat cardiomyocytes upon Dox treatment with two replicates. **J** The expression alteration of *Fosl1* in rat cardiomyocytes, mouse heart and human iPSC-derived cardiomyocytes after Dox treatment

We then investigated whether Dox_H3K27ac-upregulated genes in rats would also be upregulated in response to Dox in other species. RNA-seq data from mouse hearts treated with Dox showed that 81.6% of significantly changed genes were upregulated, while only 18.4% were downregulated (Fig. 7A, B). Similar results were observed in human iPSC-derived cardiomyocytes, where 54.6% of significantly changed genes were upregulated and 45.4% were downregulated (Fig. 7C, D). Furthermore, Dox_H3K27ac-upregulated genes also tended to be upregulated in both mouse hearts and human iPSC-derived cardiomyocytes after Dox treatment. These upregulated genes were also enriched in terms related to cardiotoxicity (Fig. 7E–H). For example, FOSL1, a pro-apoptotic protein [43, 44], displayed increased broad H3K27ac deposition across its gene locus for nearly 10 kb after Dox treatment in rat cardiomyocytes (Fig. 7I). Consistently, Dox generally increased the expression of *Fosl1* in rat cardiomyocytes, mouse hearts, and human iPSC-derived cardiomyocytes after Dox treatment (Fig. 7J). Taken together, genes regulated by H3K27ac, which are associated with cardiotoxicity, exhibited a conservative pattern of upregulation across rat, mouse, and human cardiomyocytes in response to Dox.

## Discussion

P300 is generally believed to be present in multiple epigenetic complexes with CBP, and is involved in the acetylation modification of various types of histone marks including H3K27ac [45–47]. A previous study suggested that Dox treatment induces an acute amplification of p300 level [48]. In addition, Dox treatment reduces the expression of histone deacetylases, such as HDAC2 [16], which contributes to the removal of H3K27ac [49]. These findings further support our discovery of increased H3K27ac levels following Dox treatment.

H3K27ac deposition primarily occurs at open chromatin loci, such as active promoters and enhancers, and plays a role in transcriptional activation [17, 18]. Meanwhile, super-enhancers are characterized by a high deposition of H3K27ac and are closely associated with the activation of genes involved in determining cell fate [28, 50]. BRD4 is a reader of H3K27ac that binds acetylated histones H3 via its bromodomain and recruits p-TEFb complex, which activates transcription and facilitates the elongation of mRNA [51]. Our findings demonstrate that the H3K27ac signal increased at multiple genes associated with cardiotoxicity upon Dox treatment, and this increase is accompanied by elevated mRNA expression. These results suggest that Dox-induced H3K27ac serves as a molecular switch for activating genes related to cardiotoxicity.

In the present study, we have discovered that robust apoptosis genes, including *Bax*, *Fas* and *Bnip3,* exhibit an increased H3K27ac signal at their promoters and are up-regulated following Dox treatment. These genes have reported a positive correlation between the increased expression of *Bax*, *Fas* and *Bnip3* in cardiomyocytes and the severity of Dox-induced cardiotoxicity [9, 31–33]. Therefore, Dox-induced H3K27ac deposition at genome may be a prime driver of myocardial injury. Several genes with both increased H3K27ac signal and mRNA levels, including *Egr-1, Adam17*, *Dusp6*, *Ddit4* and *Angptl4,* have been implicated in the progression of myocardial damage [34–42]. Knockout of *Adam17* specifically in cardiomyocytes has been shown to ameliorate left ventricular remodeling in diabetic cardiomyopathy and myocardial infarction in mice [36, 37]. Additionally, DDIT4 has been found to promote methamphetamine-induced cardiotoxicity through the mTOR signaling pathway in cardiomyocytes [40]. Genetic study has reported an association between variants of *Angptl4* and a reduced risk of coronary heart disease. These genes may also contribute to Dox-induced cardiotoxicity [42]. Therefore, based on the changes in H3K27ac signal across the genome and gene expression, it is possible to predict more potential therapeutic targets for Dox-induced cardiotoxicity.

Multiple signaling pathways potentially contribute to the Dox-induced cardiotoxicity. Besides the apoptotic process, genes showing increased narrow H3K27ac signal following Dox treatment were also enriched in the aging process. Previous studies have demonstrated that Dox induces cardiotoxicity by accelerating aging in cardiomyocytes [52]. One possible explanation is that Doxorubicin increases the deposition of H3K27ac on aging-associated genes, thereby promoting their expression and further accelerating aging. Furthermore, genes exhibiting increased broad H3K27ac signal following Dox treatment were also enriched in terms related to response to ischemia and hypoxia. The up-regulation of several genes in these two terms is positively correlated with cardiomyocyte injury. For instance, H3K27ac signal around the TSS of *Ppif* and *Camk2d* increased following Dox treatment. These two genes are enriched in response to ischemia and hypoxia, respectively. Previous studies have shown that *Ppif* knockdown reduces H_2_O_2_-induced cardiomyocyte death [53]. CAMK2D contributes to myocardial damage by influencing the expression of inflammatory gene [54], serves as an inducer of cardiac disease [55]. Therefore, Dox-treated hearts may exhibit a phenotype resembling ischemia and hypoxia, activating the expression of ischemia and hypoxia-responsive genes. The upregulation of certain genes in this context might exacerbate myocardial toxicity.

C646 is a histone acetyltransferase inhibitor that inhibits p300, and can reduce histone H3 acetylation levels [56, 57]. Moreover, C646 has been found to induce apoptosis in androgen-sensitive castrated prostate cancer cells by interfering with the androgen receptor (AR) and NF-κB pathway [58]. Consistent with this, we found that C646 protected mice against Dox-induced cardiotoxicity. Given that H3K27ac is a driver of Dox-induced myocardial toxicity, inhibiting H3K27ac with C646 may help reduce myocardial toxicity. Furthermore, considering the anti-tumor activity of C646, combining C646 with Dox may be an effective strategy to enhance anti-tumor activity while reducing myocardial toxicity. The molecular mechanisms underlying the response to Dox mediated by C646 in cardiomyocytes and cancer cells should be further investigated.

## Conclusions

**Fig. 8:**
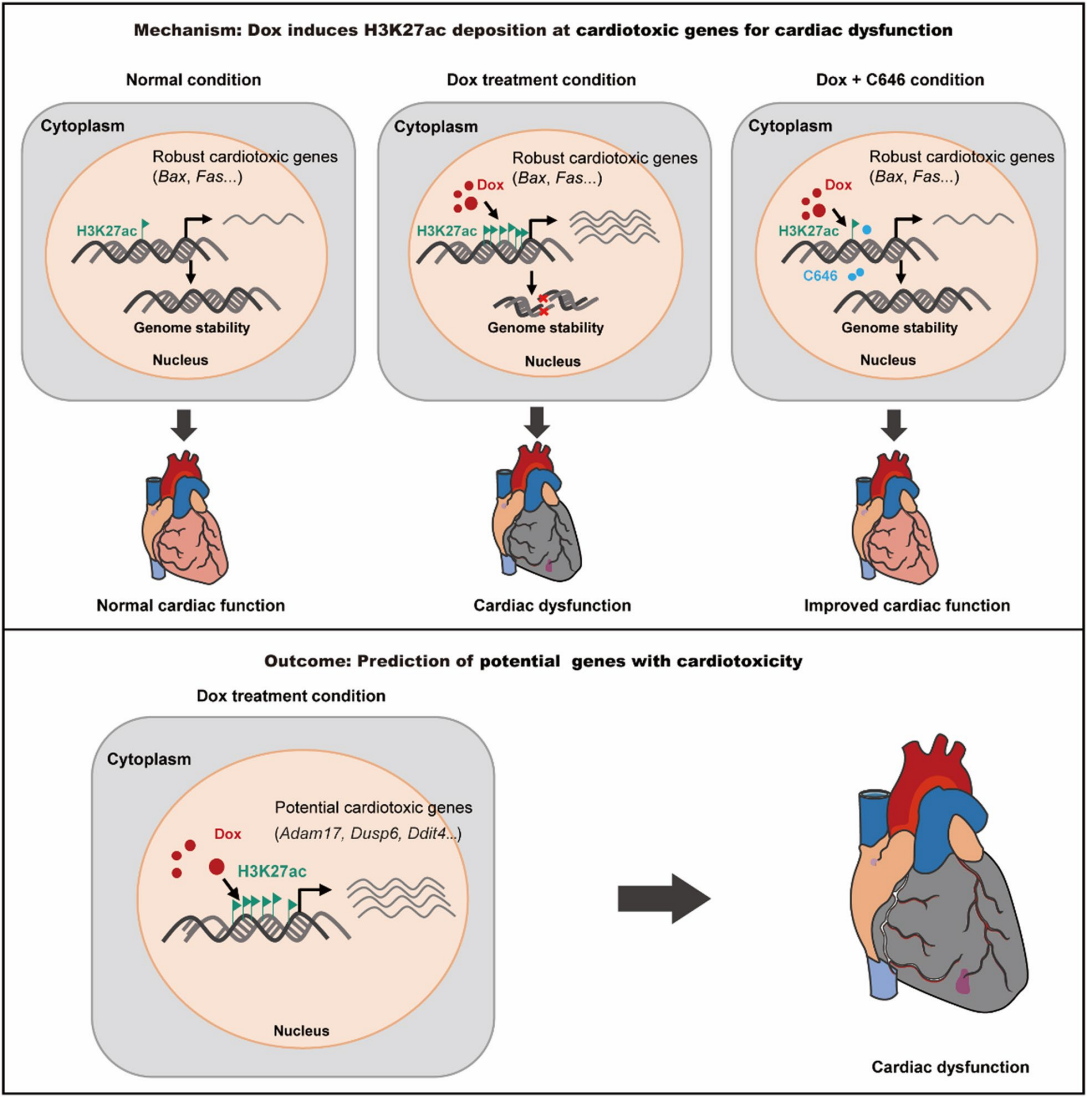
Model for the activation of robust cardiotoxic genes during doxorubicin-induced cardiotoxicity. Dox treatment increases the deposition of H3K27ac at promoters of robust cardiotoxic genes, and activates their transcription in cardiomyocytes. These results further promote DNA damage in the nucleus, and excessive genome instability leads to heart failure. C646 treatment reduces the expression of doxorubicin-induced cardiotoxic genes, and improves the cardiac function

Our findings demonstrate that Dox treatment induces broad deposition of H3K27ac at the promoters of robust apoptosis genes, leading to their activation in cardiomyocytes. These results contribute to genome instability and further impair heart function. Inhibition of H3K27ac with C646 alleviated Dox-induced DNA damage in cardiomyocytes and protected against heart dysfunction. Based on the H3K27ac deposition and mRNA expression levels, we predicted several potential therapeutic targets for treating Dox-induced cardiotoxicity (Fig. 8). Finally, cardiotoxic genes regulated by H3K27ac were conservatively up-regulated upon Dox treatment across species. Collectively, these data provided evidence for a previously unrecognized role of H3K27ac as a prime driver of Dox-induced myocardial injury, which provided insights into the relationship between epigenetics and Dox-induced cardiotoxicity.

## Methods

### Animals

C57BL/6J mice and SD neonatal rats were purchased from Laboratory Animal Center, Sun Yat-Sen University. Male mice were used for the experiments and maintained in a 12-h light/dark cycle with controlled temperature and humidity, and had free access to food and water. Usage protocols for all animals have been reviewed and approved by the Institutional Animal Care and Use Committee (IACUC), Sun Yat-Sen University (Guangzhou, China).

### Isolation and culture of cardiomyocytes

Neonatal rat cardiomyocytes (NRCMs) were isolated from the ventricles of rats (1–3 days old). Newborn rat ventricular tissue was cut into pieces and digested in 0.1% trypsin (Gibco) at 4 °C overnight for 12 h. In the next day, the ventricular tissue was digested in 0.1% Type II Collagenase (Worthington) at 37 °C for 10 min for three times. When the digested hearts were mechanically disassociated, collected the supernatant separately and mixed with equal volume of DMEM medium with 10% fetal bovine serum (FBS). The supernatant was filtered through a 70 μm mesh, and followed by centrifugation at 1500 rpm for 5 min. Cells were collected and incubated in 100 mm petri dish at 37 °C in a 5% CO2 incubator for 1 h for two times, according to different adhesion time between cardiomyocyte and fibroblast to purify the cardiomyocyte. Then the unattached cells, composed mostly of cardiomyocyte, in the suspension were collected, and cultured in DMEM medium with 10% FBS (Gibco), 1% penicillin/streptomycin for further study (Gibco).

### In vivo doxorubicin treatment

2 mg C646 was dissolved in 200 µL DMSO, which was sonicated until fully dissolved. Subsequently, 800 µL of PEG300 was added and thoroughly mixed, followed by 100 µL of Tween-80. Finally, 900 µL of physiological saline was added to achieve a final concentration of 1 mg/mL C646 solution. Doxorubicin was dissolved in saline.

Eight-week-old male mice were divided into four groups for the animal model study. Group I (Control group) served as the control, receiving weekly saline injections and vehicle injections of C646 twice a week via intraperitoneal route over 4 weeks. Group II (C646 group) received C646 injections (5 mg/kg) twice a week for 4 weeks. Group III (Dox group) received doxorubicin (Dox) injections (5 mg/kg) once a week for 4 weeks (total cumulative dose: 20 mg/kg). Group IV (Dox + C646 group) received C646 injections (5 mg/kg) twice weekly, administered 30 min prior to and 3 days following each Dox injection (5 mg/kg), over a 4-week period.

For detecting the expression of genes in Dox-treated hearts, 8-week-old male mice were randomized into 2 groups. Group I: Control group. The mice in this group received saline injection via intraperitoneal route. Group II: Dox group. In this group, the mice received Dox injection at a dose of 20 mg/kg via intraperitoneal route. After the mice were treated for 24 h, the hearts were excised for RNA extract and real-time quantitative PCR.

### Western blot

Proteins were extracted from cultured cells using RIPA lysis buffer (Beyotime) at 4 °C. Then, the protein lysates were separated by sodium dodecyl sulfate–polyacrylamide gel electrophoresis (SDS-PAGE) and transferred to polyvinylidene fluoride membranes (Millipore). After blocking with 5% BSA (Sigma) for 1 h at room temperature, the membrane was incubated with primary antibodies overnight at 4 °C. After incubated with secondary antibody, the membrane was visualized using the Bio-Rad. Antibodies against the following antigens were used: γH2AX (1:1000, cst), H3K27ac (1:1000, Abclonal), cleaved caspase3 (1:1000, cst), BCL-2 (1:1000, Abclonal), GAPDH (1:1000, Proteintech), ACTIN (1:1000, Proteintech), Acetyl-Histone H3 (1:1000, Abclonal).

### Cell counting Kit-8 assay

Cell counting kit-8 (Elabscience) was used to assess cell viability according to the manufacturer’s instructions. In brief, NRCMs were prepared at a concentration of 30,000 cells/mL. The cell suspension (100 μL/well) was seeded in 96-well plates. Then, CCK-8 solution (10 μL/well) was added for further detection. After incubation at 37 °C for 2–4 h, absorbance was measured at 450 nm.

### Cell immunofluorescence staining

Cells in different groups were fixed in 4% paraformaldehyde for 10 min, followed by permeabilizing and blocking in Immunol Staining Blocking Buffer (Beyotime) for 1 h. Then, cells were incubated with primary antibody (1:200–1:500) diluted in Immunol Staining Primary Antibody Dilution Buffer (Beyotime) overnight at 4 ˚C. After three times washes with PBST, cells were then incubated in fluorescent secondary antibody (1:500) diluted in Immunol Fluorescence Staining Secondary Antibody Dilution Buffer (Beyotime) for 1 h at room temperature following washes with PBST three times. Finally, the cells were blocked with Antifade Mounting Medium with DAPI. A1R N-SIM N-STORM microscope was used to visualize the staining. Representative images were selected based on their quality and most accurately represent the group mean across all the available data. NIS-Elements software (version 4.60.00, Nikon) was used for visualization of the stained samples, imaris software (version 10.0.0, Bitplane) was used for image analysis.

### TUNEL staining

To quantify apoptosis, the TUNEL assay was performed using a One-step TUNEL In Situ Apoptosis Kit (Elabscience) according to the manufacturer’s instructions. A1R N-SIM N-STORM microscope was used to capture the images of cells. Imaris software was used to measure the percentage of TUNEL-positive cells.

### Animal echocardiography

Cardiac function was evaluated in mice anesthetized with 2% isoflurane by transthoracic echocardiography (VisualSonics VeVo 3100 Imaging System, Toronto, Canada). Keep the body temperatures between 36.9 and 37.3 °C and the heart rates at 400–550 bpm, ejection fraction and fractional shortening for each mouse were measured by an investigator blind to the allocation of treatment.

### RNA extraction and quality control

Total RNA from each tissue was extracted using a mRNA Isolation Kit (Promega). After purified and treated with DNase, RNA purity was measured using a NanoDrop One spectrophotometer (Thermo Fisher Scientific). The total mRNA was extracted for sequencing. Each group was prepared with three replicates.

### CUT&Tag

CUT&Tag kit (YESEN) was used to detect H3K27ac binding DNA according to the manufacturer’s instructions. Briefly, neonatal rat cardiomyocytes (NRCMs) cultured in DMEM medium with 10% FBS (Gibco) were collected gently in PBS to performed for the CUT&Tag of H3K27ac. The bead slurry resuspended in ConA bind buffer were added to the cell suspension. After inverting 5 times to mix, place the PCR tube on the rotator and mix for 10 min. Then H3K27ac primary antibody was added to the cell suspension. After gently vortex, samples were incubated with primary antibody overnight at 4 °C. After removing the primary antibody, second antibody was added and incubated with rotation for 2 h at room temperature. Then 49 μL BF3-Tag buffer and 1 μL pA/G-Transposome were added. After gently vortex, samples were incubated with rotation for 2 h at room temperature. Then removing the tube from the magnetic stand and adding 30 μL BF3-Tag buffer and 1 μL 30× Activating buffer. After removing the tube from the rotator and spinning the tube briefly (< 100×*g*), 2 μL 15× Terminate Solution and 1 μL 30× Proteinase K were added and mixed thoroughly by vigorously vertexing. Then the samples were incubated at 55ºC for 30 min with thermocycler. The DNA were collected and purified with DNA Selection Beads for library amplification in the PCR program.

### CUT&Tag data analysis

Fastq files were quality-checked by trim_galore (version 0.5.0) and aligned onto Rnor_6.0 reference genome using Bowtie2 (version 2.5.1) with default parameters. Samtools (version 1.17) was used to discard the duplicates from mapped reads, then MACS2 (version 2.2.9.1) was used to call peaks.

### RNA-seq data analysis

Fastq data were quality-checked by trim_galore (version 0.5.0, default parameters), and the paird-end reads were aligned onto Rnor_6.0 reference genome using STAR (version 2.7.9a, default parameters). Htseq-count (version 2.0.1) was used to calculate gene-specific read counts, then the differentially expressed genes were calculated.

### Statistical analysis

Quantitative data were demonstrated as means ± SD. A two-tailed Student’s t-test was used to analyze the difference between two groups unless specifically stated in the legends. The differences were considered to be statistically significant, when *P < 0.05, **P < 0.01 and ***P < 0.001.

## Data Availability

Sequence data generated in this study have been uploaded to the NCBI (Accession Numbers: PRJNA1065128 and PRJNA1117743).

## Abbreviations

NRCMs: Neonatal rat cardiomyocytes
Dox: Doxorubicin
HDACs: Histone deacetylases
WT: Wild-type
EF: Ejection fraction
FS: Fractional shortening
LVIDs: Left ventricular internal diameter at end systole
LVVs: Left ventricular volume at end systole
LDH: Lactate dehydrogenase
CKMB: Creatine kinase-MB
CTNT: Cardiac troponin T
